# Long-Term Course to Lumbar Disc Resorption Patients and Predictive Factors Associated with Disc Resorption

**DOI:** 10.1155/2017/2147408

**Published:** 2017-07-09

**Authors:** Jinho Lee, Joowon Kim, Joon-Shik Shin, Yoon Jae Lee, Me-riong Kim, Seon-Yeong Jeong, Young-jun Choi, Tae Kyung Yoon, Byung-heon Moon, Su-bin Yoo, Jungsoo Hong, In-Hyuk Ha

**Affiliations:** Jaseng Spine and Joint Research Institute, Jaseng Medical Foundation, Seoul, Republic of Korea

## Abstract

The long-term course to lumbar intervertebral disc herniation (LDH) patients receiving integrative Korean medicine treatment and predictive factors associated with disc resorption were investigated. LDH patients who received integrative Korean medicine treatment from February 2012 to December 2015 and were registered in the “longitudinal project for LDH on MRI” were included. Disc resorption amount was measured 3-dimensionally with disc degeneration and modic change levels on baseline and follow-up MRIs. Patient characteristics, Korean medicine use, pain, symptom recurrence, and satisfaction were assessed through medical records and phone surveys. Of 505 participants, 19 did not show disc resorption, while 486 did. A total of 220 displayed resorption rates of ≥50%. LDH volume at baseline was 1399.82 ± 594.96 mm^3^, and that on follow-up MRI was 734.37 ± 303.33 mm^3^, indicating a 47.5% decrease (*p* < 0.0001). Predictive factors for disc resorption were disc extrusion, Komori migration classification, and LDH amount. Approximately 68.4% did not experience symptom recurrence over the 51.86 ± 19.07-month follow-up, and 90.3% were satisfied with Korean medicine treatment. The majority of LDH patients who improved after integrative Korean medicine treatment showed disc resorption within 1 year with favorable long-term outcomes. Predictive factors for disc resorption should be duly considered for informed decision-making. This trial is registered with ClinicalTrials.gov NCT02841163.

## 1. Introduction

Recent developments in the modernization of traditional Oriental medicine include greater integration with medical technology and devices toward greater efficacy, safety, and diagnostic and prognostic accuracy. A dual medical system of conventional medicine and traditional Korean medicine is employed in Korea, and conventional diagnostic imaging such as X-rays and magnetic resonance imaging (MRI) and advances in technology may be used to further promote the modernization of traditional Oriental medicine by means of an integrative and collaborative approach.

Prevalence estimates for sciatica have been reported to range from 1.6% in the general population to 43% in select working populations, and the most commonly cited cause of sciatica is lumbar intervertebral disc herniation (IDH) [1]. Low back pain (LBP) and sciatica incur most time off work and disability of all medical conditions [2], and LBP from spinal diseases such as spondylosis and intervertebral disc disorders were the 2nd highest ranking reason for hospitalized care in the working population (age 18–44) in the US in 2005 [3]. Approximately 317,000 lumbar surgeries were performed in the US in 1997 [4], which has steadily risen to 1 million spinal procedures in 2002 [5].

However, the natural history of lumbar IDH is favorable, and 70% of patients recover from sciatica without surgery within 6 weeks [6]. The 10-year observation results of surgical and nonsurgical treatment show no significant difference after 4 years [7], and a recent large-scale study on acute IDH reported that while early surgical intervention may be more beneficial economically through swift recovery and return to work, 1-year pain and function results did not differ significantly from nonsurgical treatment [8]. An estimated 10% of lumbar IDH patients consider surgery due to continuous pain and/or neurological deficit, and accurate prediction of development into chronic pain is therefore of importance [6]. Advances in radiological examination have revealed how IDH may be spontaneously resorbed in time with resolution of neurological symptoms [9], and various factors predictive of disc resorption are being studied as they hold valuable information on chronicization and need for surgical intervention [10–16].

In Korea, many IDH patients opt for primary care at Korean medicine institutions to receive Korean medicine treatment and avoid surgery. The authors have previously reported the long-term outcomes and risk factors for poor prognosis in lumbar IDH patients receiving integrative Korean medicine treatment, excluding conventional treatment [17, 18]. The objectives of this study were to observe the long-term course to IDH patients with spontaneous disc resorption after integrative Korean medicine treatment and identify predictive factors associated with resorption amount.

## 2. Materials and Methods

Jaseng Hospital of Korean Medicine, certified by the Korean Ministry of Health and Welfare as a spine-specialty Korean medicine hospital, operates 19 branches which treat 900,000 spinal disorder patients per year [19]. Electronic medical records (EMRs) and radiological assessments of participants who were diagnosed with lumbar IDH on MRI, received integrative Korean medicine treatment at these medical institutions from February 2012 to December 2015, and were included in the “longitudinal project for IDH on MRI” were assessed. The aim of the project was to assess differences in diagnostic imaging before and after treatment by allowing physicians to select 1-2 patients annually for complimentary follow-up MRIs with written consent for use of data for academic means. Survey assessments were conducted by phone from April to October 2016. This study received approval from the Institutional Review Board of Jaseng Hospital of Korean Medicine (JASENG 2016-06-003).

### 2.1. Participants

The inclusion criteria were as follows: (1) patients diagnosed with lumbar IDH on MRI, (2) patients who experienced improvement of LBP and/or sciatica from integrative Korean medicine treatment, and (3) written consent to participate in the “longitudinal project for IDH on MRI” and a follow-up MRI. The exclusion criteria were (1) patients who received lumbar spinal surgery during integrative Korean medicine treatment, (2) patients with main complaint of cervical IDH, and (3) patients with MRI errors or low resolution.

### 2.2. EMR Assessment and Phone Survey

EMRs were reviewed to assess participant characteristics including sex, age, LBP and/or sciatica, and details of integrative Korean medicine treatment (whether the patient underwent hospitalization, days in inpatient and ambulatory care, and frequency of treatment). Phone surveys were also conducted to investigate initial and current levels of pain associated with lumbar IDH as assessed by the numeric rating scale (NRS), whether the patient received recommendation for surgery, experienced recurrence of symptoms, or underwent lumbar surgery, awareness of spontaneous disc resorption, and satisfaction with Korean medicine treatment.

### 2.3. Analysis of Imaging Results

This study used three-dimensional measurements on MRI to determine IDH amount according to previous measurement methods [10]. The authors selected a main IDH level on sagittal sections of T2-weighted MRIs best correlating with symptoms. A line connecting 2 points indicating the posterior edges of the superior and inferior endplates was drawn as reference. The IDH area and amount outside the reference line were demarcated and measured using the “measure area freehand” function in the picture archiving and communication system (PACS) on sagittal sections. The volume was calculated by multiplying the IDH area on each sagittal section with the MRI scan thickness plus the interslice intervals.

The IDH level was classified into bulging, protrusion, extrusion, migration, and sequestration and by the migration criteria suggested by Komori et al. [20] where migrating disc amount is categorized in comparison to the posterior height of the adjacent vertebrae. IDH < 1/3 of the posterior height of the vertebrae immediately superior or inferior to the IDH level was designated as Grade 2, that between 1/3 and 2/3 height as Grade 3, >2/3 height as Grade 4, and no IDH as Grade 1. The IDH degeneration categorization employed the classification criteria put forth by Pfirrmann et al., which classifies degeneration by structure, signal intensity, and disc height on MRI into 5 levels from Grades I to V. Higher grades imply greater degeneration, and generally Grades IV and V are taken to indicate disc degeneration [21]. Modic types were classified into 0 (no modic change), and modic types 1, 2, and 3. The location of modic type change in the vertebral body was recorded as (i) above, (ii) below, or (iii) both above and below the IDH level, or (iv) no modic type change. Two Korean medicine doctors (KMDs) experienced in spinal imaging received training for standardized reading for higher reliability. Interrater reliability was assessed through measurement and classification of 20 random pairs of imaging results (total of 40 MRIs before and after treatment) (see Supplementary Table  1 in Supplementary Material available online at https://doi.org/10.1155/2017/2147408).

### 2.4. Statistical Analysis

All continuous variables are expressed as mean ± SD, and categorical variables as *n* (%). Consistency of IDH imaging measurement was determined through intraclass correlation coefficient (ICC), and that of IDH and degeneration classification was analyzed by Cohen's kappa. Predictive factors for ≥50% IDH regression on follow-up MRI were determined through logistic regression analysis considering for major influence variables (age, gender, level of disc degeneration, IDH type, Komori migration classification, modic type, modic change area, time interval between baseline and follow-up MRIs, and total treatment duration). Univariate analysis was performed, and factors that were significant in univariate analysis were included in additional multivariate analyses with age and baseline IDH amount, adjusted for. Comparison of IDH amounts before and after treatment was analyzed using paired *t*-test and IDH type and level of degeneration were analyzed with chi-square test. Associations between IDH volume on baseline and follow-up MRIs, change in volume, resorption rate, and age were assessed through regression analysis. All analyses comparing measurements before and after treatment were performed using STATA 14.0 (StataCorp, College Station, Texas, USA).

## 3. Results

**Figure 1:**
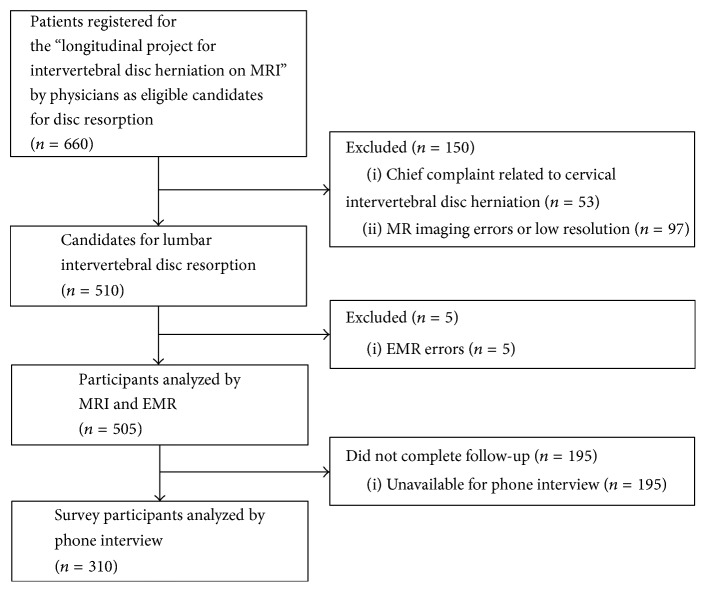
Flow diagram of the study.

A total of 660 patients were registered in the “longitudinal project for IDH on MRI” and underwent baseline and follow-up MRIs. Of these eligible participants, patients with chief complaint diagnosis of cervical IDH, imaging or EMR errors, and low imaging resolution were excluded, and 505 lumbar IDH patients participated. Phone surveys were performed in all participants except those that the researchers were unable to reach, yielding a total of 310 survey participants (61%) (Figure 1).

Baseline demographic characteristics of the 505 participants were as follows: Average age was 39.08 ± 10.19 years, with higher male percentage (60.6%), and the most common IDH level was L4/5 (53.3%) and L5/S1 (38.8%). The majority did not have LBP (90.1%), and most presented with sciatica (83.6%) which was mainly unilateral (76.0%) (Table 1).

The average time interval between baseline and follow-up MRI was 341.38 ± 306.83 days, and the difference between MRIs was analyzed. Evidence of disc resorption was not seen in 19 patients and seen in 486 of whom 220 exhibited a resorption rate of ≥50%. The IDH volume at baseline was 1399.82 ± 594.96 mm^3^, and that at follow-up was 734.37 ± 303.33 mm^3^, indicating a statistically significant volume decrease of 47.5% (*p* < 0.0001). In Komori migration classification, 38.6% of patients were classified as Grade 2 or 3 at baseline, which decreased to 3.4% at follow-up. Regarding IDH type, the percentage of patients with extrusion or greater herniation level (i.e., extrusion, migration, or sequestration) at baseline was 87.9%, which declined to 22.8% at follow-up. Disc degeneration and modic type grade did not show significant difference (Table 2).

Of the 505 participants, 186 (38.6%) were hospitalized while the others received integrative Korean medicine treatment in the outpatient department. The number of days in inpatient care was 34.34 ± 29.53 days, that of outpatient visits was 32.88 ± 20.72 days, and the total number of days in care was 45.53 ± 28.61 days. The vast majority of patients were given herbal medicine (96.4%), acupuncture (96.4%), and Chuna manipulation (87.9%), followed by bee venom pharmacopuncture (65.0%), pharmacopuncture (53.3%), electroacupuncture (46.5%), and cupping (22.6%). Of herbal medicine and pharmacopuncture, Chungpa-jun and variations, of which GCSB-5 is the main ingredient [22–25], and Shinbaro pharmacopuncture [26] were used most frequently, respectively (Table 3).

The mean time interval between baseline and follow-up phone interviews in the 303 survey participants was 51.86 ± 19.07 months. Baseline pain NRS was reported at 8.34 ± 1.42, which was put at 1.27 ± 1.57 for current pain. Lumbar surgery had previously been recommended for around half of all patients at baseline (54.2%), and 73.2% were not aware of spontaneous disc resorption. Ninety-four patients reported pain recurrence(s) during the follow-up period (30.3%), and the majority chose Korean medicine for its treatment. Most patients were satisfied with Korean medicine (90.1%) and nonsurgical methods for lumbar IDH treatment were recommended for them (94.2%) (Table 4).

**Figure 2:**
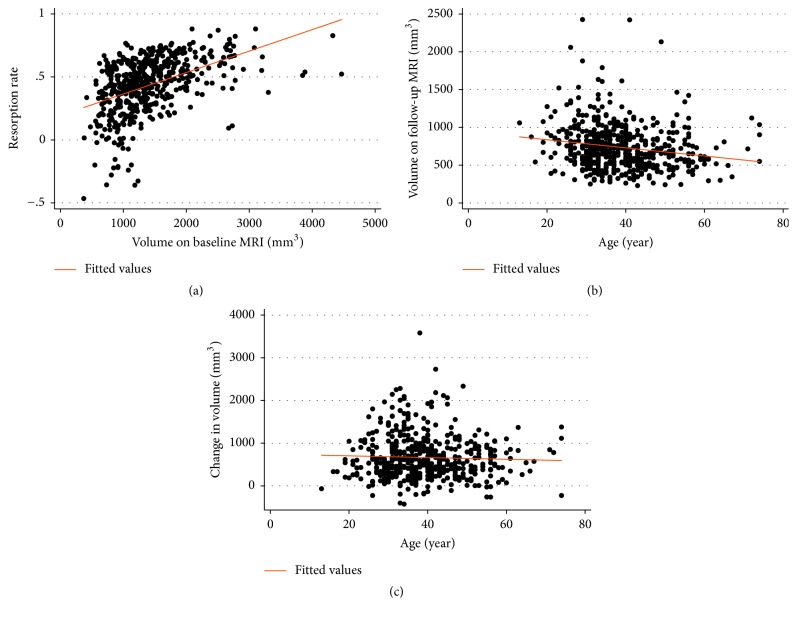
Associations between herniated disc volume on baseline MRI, volume on follow-up MRI, change in volume, disc herniation resorption rate, and age. (a) Association between herniated disc volume on baseline MRI and resorption rate. *y* = 0.19 + 0.0001703*x*, *R*^2^ = 0.2148, *p* ≤ 0.001. (b) Association between herniated disc volume on follow-up MRI and age. *y* = 944.39 − 5.37*x*, *R*^2^ = 0.0326, *p* ≤ 0.001. (c) Association between change in herniated disc volume and age. *y* = 744.62 − 2.03*x*, *R*^2^ = 0.0016, *p* = 0.362.

Analysis of predictive factors for ≥50% resorption (dichotomous variable) in IDH resorption amount revealed that IDH type and amount (Komori migration classification) were significantly associated. When Komori migration classification Grade 1 was set as reference, the odds ratios (ORs) for Grades 2 and 3 were 1.7 (95% CI 1.04–2.76) and 2.46 (95% CI 1.37–4.42), respectively. When protrusion was designated as the reference in IDH type, the ORs for extrusion and migration were 2.49 (95% CI 1.10–5.60) and 6.3 (95% CI 2.58–15.42), respectively (Table 5). Baseline IDH amount was shown to be positively related to disc resorption rate (continuous variable), whereas age was negatively associated (Figure 2).

## 4. Discussion

Of 505 participants who were mainly middle-aged males presenting with unilateral sciatica symptoms due to IDH at L4/5 and L5/S1 levels, 486 displayed spontaneous disc resorption (96.2%), and 220 showed resorption rates of ≥50% (43.6%). In Komori migration classification, 38.6% of patients were classified as Grades 2 or 3 at baseline which decreased to 3.4% at follow-up, and 87.9% were classified as disc extrusion or higher herniation level on baseline MRI, compared to 22.8% at follow-up regarding IDH type. Major factors determined to predict disc resorption were baseline IDH type, Komori migration classification, and age. Over a 51.86 ± 19.07-month follow-up period, 68.4% did not experience LBP recurrence, and 90.3% replied that they were satisfied with Korean medicine treatment.

The underlying mechanism of disc resorption has been suggested to be enzymatic degradation and phagocytosis of IDH matter through inflammatory reaction and vascularization. As IDH material enters the vascularized epidural space, it is identified by the body as a foreign substance, leading to immune and inflammatory response, and thus induces neovascularization, enzymatic degradation, and macrophage phagocytosis. This consequently leads to production of matrix proteinases and increased cytokine levels, resulting in disc resorption [27, 28].

The results of this study are consistent with other studies where sequestrated disc matter was shown to be better resorbed than protruded disc. This is speculated to be due to the degree of penetration (tear) of the annulus fibrosus and posterior longitudinal ligament and increased exposure to systemic circulation within the epidural space [29, 30].

A recent systematic review on the probability of spontaneous disc resorption by IDH type including 9 papers covering 361 patients reported that 96% of disc sequestration (resorption in 52/54 patients), 70% of disc extrusion (108/154), 41% of disc protrusion (38/93), and 13% of disc bulging types (8/60) were resorbed, indicating high resorption rates in sequestration and extrusion types [12]. Similarly, Komori et al. reported that although 78% of migrated discs resulted in resorption (resorption in 36/64 patients), only 17% (7/48) were resorbed in nonmigrated discs [20], and went on to publish that whereas the complete resolution rate of migrated discs was 41%, that of nonmigrated discs was 0% [31]. In a study by Ahn et al., 25 out of 36 discs showed reduction in size, where 56% of subligamentous, 79% of transligamentous, and 100% of sequestered disc herniation resulted in IDH size reduction, and the study concluded that transligamentous extension and posterior longitudinal ligament rupture were the most influential factors in IDH reduction [29]. Moreover, in a 2014 observational study comparable to the current study on 102 patients receiving traditional Chinese medicine treatment, 81.37% reported symptom improvement and 18.63% were considered to require surgery. IDH volume decreased from 1433.89 ± 525.49 mm^3^ to 1002.01 ± 593.95 mm^3^, resulting in an average resorption rate of 27.25 ± 32.97%, and 20 patients presented resorption rates of ≥50% [10]. While modic change was not recognized as a predictive factor of resorption in this study, a 2014 study by Shan et al. reported that whereas 35 of 85 patients in the modic change group (of whom the majority were of Type 2) did not show much difference in IDH size, the group with no modic change displayed significant decrease [32]. Iwabuchi et al. purported that resorption factors could be identified on T1- and T2-weighted MRIs, with high signal intensity of IDH areas in T1- and T2-weighted MRIs in the nonregression group [33].

The largest strength of this study is that, to the best of our knowledge, it covers the largest sample size (*n* = 505) of studies addressing disc resorption. This is especially important in research on predictive factors for disc resorption as it is difficult for small sample sized studies to secure sufficient statistical power. Furthermore, while predictive factor studies should consider various factors through prediction models using multivariate analysis, only univariate analysis may be possible in studies with small sample size. Another major strength of this study is that IDH was measured 3-dimensionally on MRI. Two-dimensional cross-sectional images may differ greatly from positioning, and various limitations and errors arise in measurement from the sectional directions in image acquisition. This study also includes long-term observation results at an average of 51.86 months (approximately 4.3 years) in addition to imaging analyses to better portray current state, symptom recurrence, and satisfaction with treatment.

Despite these strengths, this study also suffers from the following weaknesses. The largest limitation is probably the fact that although the “longitudinal project for IDH on MRI” itself was conducted prospectively, outcome measures were partly investigated in a retrospective manner through EMR examination and include limited clinical information. Moreover, although follow-up MRIs were conducted allowing for sufficient time for potential disc resorption as clinical settings and timeframes permitted, the time intervals are inconsistent as a result. The fact that MRI scans were performed using different imaging apparatus and under different conditions at various in-hospital and external sites in this multicenter study may also be viewed as a limitation.

While the natural course of lumbar IDH is widely considered to be favorable, surgical interventions are still common [4, 5]. International consensus recommends consideration of surgery if symptoms persist after a certain period of conservative treatment [34]; however, consensus has not been reached regarding its duration [35, 36]. Although severe IDH potentially incurs neurological disability and higher pain levels, it also entails greater possibility of disc resorption. A recent trial on recovery of motor deficit which is widely considered to require surgical attention reported that differences between early surgery and prolonged conservative treatment at 1 year were nonsignificant [37]. Early surgery in lumbar IDH patients should be approached carefully and relevant information shared with patients in the decision-making process. Despite the fact that most participants were recommended for surgery in this study, not many were aware of spontaneous disc resorption.

Prospective large-scale studies further examining the disc resorption prediction process are warranted. Risk scores such as the Framingham Risk Score used to estimate 10-year cardiovascular risk may be similarly proposed in IDH through development of a prediction model to provide patients, physicians, and healthcare givers with valuable data necessary for informed decision-making in selecting IDH treatment.

## 5. Conclusion

In conclusion, the majority of patients who received conservative integrative Korean medicine treatment indicated a disc resorption volume of nearly 50% within a 1-year average, and the long-term course at 4.3 years was also favorable. IDH amount and type were identified as predictive factors associated with disc resorption and this information should be factored into prognosis and informed decision-making in treatment selection as most patients were unaware of the fact that disc resorption may occur spontaneously.

## Supplementary Material

Interrater reliability of MRI volume measurement, and grade and type classification.

## Figures and Tables

**Table 1:** Baseline demographic characteristics of participants of the “longitudinal project for IDH on MRI”.

Variables	*n* = 505		
	*n*	%	Mean (SD)
Age (years)			39.08 ± 10.19
<25	23	4.6	
≥25–<55	436	86.3	
≥55	46	9.1	
Sex			
Male	306	60.6	
Female	199	39.4	
Herniated disc level			
L1/2	2	0.4	
L2/3	9	1.8	
L3/4	29	5.7	
L4/5	269	53.3	
L5/S1	196	38.8	
Low back pain			
No	454	90.1	
Yes	48	9.5	
Radiating leg pain			
No	80	15.8	
Yes, radiating to thigh	127	25.2	
Yes, radiating to calf	239	47.3	
Yes, but with no indication of area	56	11.1	
Unknown	3	0.6	
Bilateral radiating leg pain			
No	432	85.5	
Yes	66	13.1	
Unknown	7	1.4	
Unilateral radiating leg pain			
No	115	22.8	
Yes	384	76.0	
Unknown	6	1.2	

IDH, intervertebral disc herniation; MRI, magnetic resonance imaging; SD, standard deviation.

**Table 2:** Baseline and follow-up MRI results.

Variables	Baseline MRI		Follow-up MRI		*p* value
	*n*	%	*n*	%	
Volume of herniated disc (mm^3^)^a^	1399.82 ± 594.96		734.37 ± 303.33		0.000
Time interval between baseline and follow-up MRIs (days)	341.38 ± 306.83				
Resorption rate (%)					
Aggravation	19	3.76			
>0–≤25	75	14.85			
>25–≤50	191	37.82			
>50–≤75	205	40.59			
>75–≤100	15	2.97			
Komori migration classification^b^					
1	310	61.4	488	96.6	0.000
2	106	21.0	16	3.2	
3	89	17.6	1	0.2	
Disc herniation type^b^					
Bulging	1	0.2	87	17.2	0.000
Protrusion	60	11.9	303	60.0	
Extrusion	328	65.0	100	19.8	
Migration	93	18.4	12	2.4	
Sequestration	23	4.6	3	0.6	
Disc degeneration grade^b^					
I	6	1.2	4	0.8	0.582
II	47	9.3	39	7.7	
III	215	42.6	202	40.0	
IV	224	44.4	243	48.1	
V	13	2.6	17	3.4	
Modic change type of vertebral body^b^					
0	358	70.9	353	69.9	0.930
1	16	3.2	14	2.8	
2	128	25.3	134	26.5	
3	3	0.6	4	0.8	
Modic change of vertebral body area^b^					
Above the herniated disc	22	4.4	20	4.0	0.981
Below the herniated disc	20	4.0	20	4.0	
Both above and below the herniated disc	105	20.8	109	21.6	
None	358	70.9	356	70.5	

^a^Paired *t*-test was used in analysis of change in amount of herniated disc matter from baseline; ^b^Chi-square test was used in analysis of change in classification of herniated disc from baseline; MRI, magnetic resonance imaging.

**Table 3:** Use of integrative Korean medicine treatment.

Variables	*n* = 505		
	*n*	%	Days (mean ± SD)
Hospitalized care	186	36.8	
Number of hospitalized days			34.34 ± 29.53
Number of outpatient visit days			32.88 ± 20.72
Total number of treatment days			45.53 ± 28.61
Frequency of integrative Korean medicine treatment			
Herbal medicine	487	96.4	136.35 ± 90.81
Pharmacopuncture	269	53.3	31.15 ± 28.62
Bee venom pharmacopuncture	328	65.0	26.78 ± 20.38
Chuna manipulation	444	87.9	39.06 ± 35.08
Acupuncture	487	96.4	45.42 ± 32.79
Electroacupuncture	235	46.5	32.80 ± 22.42
Cupping	114	22.6	20.00 ± 17.81

SD, standard deviation.

**Table 4:** Outcome measures assessed through phone interview.

Variables	*n* = 310		
	*n*	%	Mean ± SD
Time interval between initial visit and phone interview (months)			51.86 ± 19.07
NRS of pain at baseline^a^			8.34 ± 1.42
NRS of current pain			1.27 ± 1.57
Recommendation of surgery^a^			
Recommended for surgery	168	54.2	
Not recommended for surgery	112	36.1	
Do not know	6	1.9	
Did not visit conventional medicine institution	24	7.7	
Awareness of possibility of disc resorption^a^			
Aware	83	26.8	
Unaware	227	73.2	
Experience of symptom recurrence^a^			
Yes	94	30.3	
No	212	68.4	
Do not know	4	1.3	
Treatment type used for recurrence^a^			
Korean medicine treatment	104	36.1	
Conventional nonsurgical treatment	14	4.9	
Surgery	7	2.4	
No recurrence requiring treatment	163	56.6	
Satisfaction with Korean medicine treatment			
Very satisfied	159	51.5	
Satisfied	120	38.8	
Slightly satisfied	28	9.1	
Unsatisfied	1	0.3	
Very unsatisfied	1	0.3	
Recommendation of treatment to others			
Surgical treatment	4	1.3	
Nonsurgical treatment	292	94.2	
Do not know	14	4.5	
Effective Korean medicine treatment type			
Herbal medicine	64	20.7	
Bee venom pharmacopuncture, pharmacopuncture	135	43.6	
Acupuncture	26	8.4	
Chuna manipulation	60	19.4	
Do not know	61	19.7	

^a^Baseline timepoint was assessed retrospectively at the time of follow-up survey; SD, standard deviation; NRS, numeric rating scale.

**Table 5:** Assessment of predictive factors at baseline associated with herniated disc resorption in participants.

	Univariate		Multivariate^a^	
	OR	95% CI	OR	95% CI
Age (continuous)	1.01	(0.99, 1.03)		
Sex, male (ref. female)	0.97	(0.67, 1.37)		
Disc degeneration grade (ref. I)				
II	3.39	(0.37, 31.38)		
III	3.14	(0.36, 27.38)		
IV	4.57	(0.53, 39.77)		
V	16.67	(1.36, 204.03)		
Disc herniation type (ref. protrusion)				
Bulging	—			
Extrusion	4.66	(2.15, 10.13)	2.49	(1.10, 5.60)
Migration	11.82	(5.02, 27.85)	6.3	(2.58, 15.42)
Sequestration	12.190	(3.91, 37.95)	3	(0.84, 10.68)
Komori migration classification (ref. 1)				
2	2.48	(1.58, 3.89)	1.7	(1.04, 2.76)
3	5.46	(3.24, 9.18)	2.46	(1.37, 4.42)
Modic change type of vertebral body (ref. 0)				
1	0.84	(0.30, 2.37)		
2	4.4	(0.94, 2.10)		
3	0.7	(0.06, 7.81)		
Modic change of vertebral body area (ref. none)				
Above the herniated disc	0.94	(0.37, 2.35)		
Below the herniated disc	1.410	(0.57, 3.46)		
Both above and below the herniated disc	1.38	(0.90, 2.12)		
Time interval between MRIs (continuous)	1	(0.99, 1.00)		
Total treatment duration (continuous)	1	(0.99, 1.00)		

^a^Adjusted for age and amount of disc herniation; OR, odds ratio; CI, confidence interval; MRI, magnetic resonance imaging.
