# Supplementary material for: Long-Term Course to Lumbar Disc Resorption Patients and Predictive Factors Associated with Disc Resorption
Source: Evid Based Complement Alternat Med. 2017 Jul 9;2017:2147408. doi: 10.1155/2017/2147408 (PMC5523460; doi:10.1155/2017/2147408)
Supplement: Supplementary file 1 — Interrater reliability of MRI volume measurement, and grade and type classification. [file 2147408.f1.docx]

**Supplementary Table 1. Interrater reliability of MRI measurement and classification.**

| Intraclass correlation coefficient | mean | 95% CI | p value |
| --- | --- | --- | --- |
| Total volume on baseline MRI | 0.91 | (0.86, 0.94) | 0.00 |
| Total volume after follow-up MRI | 0.94 | (0.92, 0.96) | 0.00 |
| Kappa coefficient | Baseline MRI | Follow-up MRI |  |
| Disc degeneration grade | 0.66 | 0.74 |  |
| Disc herniation grade | 1.00 | 1.00 |  |
| Komori migration classification | 0.73 | - |  |
| Modic change type of vertebral body | 1.00 | 1.00 |  |
| Modic change of vertebral body area | 1.00 | 1.00 |  |

MRI, magnetic resonance imaging; CI, confidence interval
